# Abdominal complications due to collapse of a large mesenteric hematoma after rupture of a right colic artery aneurysm: a case report

**DOI:** 10.1186/s40792-021-01319-z

**Published:** 2021-10-30

**Authors:** Taro Ikeda, Masaaki Mitsutsuji, Takuya Okada, Isamu Yamada, Ryunosuke Konaka, Yukari Adachi, Akiko Matsumoto, Takahiro Wada, Naoki Harada, Masahiro Samizo

**Affiliations:** 1 Department of Surgery, Sanda City Hospital, 3-1-1 Keyakidai, Sanda, Hyogo 669-1321 Japan; 2 Department of Radiology, Kobe University Graduate School of Medicine, Kobe, Japan; 3 Division of Disaster and Emergency Medicine, Department of Surgery Related, Kobe University Graduate School of Medicine, Kobe, Japan

**Keywords:** Mesenteric hematoma, Right colic artery aneurysm, Small bowel obstruction, Cholestasis

## Abstract

**Background:**

Non-traumatic mesenteric hematomas are usually well controlled, with no resulting symptoms. Herein, we report a case in which collapse of a large mesenteric hematoma, after rupture of a right colic artery aneurysm, caused small bowel obstruction and rapid absorption of the hematoma contributed to cholestasis.

**Case presentation:**

A-44-year-old man presented with a sudden onset of severe right lower abdominal pain. Computed tomography (CT) revealed rupture of a right colic artery aneurysm and intra-abdominal bleeding. After embolization of the right colic artery aneurysm, a large mesenteric hematoma remained. As the patient had no symptoms, we elected to pursue conservative treatment. However, on day 16 post-onset, he developed right lower abdominal pain. On CT imaging, partial collapse of the wall of the residual mesenteric hematoma was observed, with visible leakage from the hematoma into the abdominal cavity, resulting in small bowel obstruction and cholestasis. Symptoms did not improve with conservative treatment, and we proceeded to surgical treatment on day 32 after onset. Intra-operatively, adhesions between the small bowel and the abdominal wall were identified and caused the small bowel obstruction. We proceeded with removing these adhesions and as much of the hematoma as possible. Although the small bowel obstruction improved after surgery, cholecystitis developed, and percutaneous transhepatic gallbladder aspiration was performed on day 45. The patient was discharged on day 70.

**Conclusions:**

Collapse of a mesenteric hematoma can cause small bowel obstruction. Rapid absorption of the hematoma due to the collapse might contribute to cholestasis. A large abdominal hematoma might be a risk factor for failure of conservative treatment, and surgery might be required due to abdominal complications.

## Background

Non-traumatic mesenteric hematoma can result from rupture of an abdominal aneurysm or as a complication of anticoagulation therapy, among other causes [1, 2]. In some cases, however, the etiology of a mesenteric hematoma is unknown [3, 4]. Although the hemorrhaging in non-traumatic mesenteric hematomas is usually well controlled with no resulting symptoms, complications can develop in some cases [3, 4]. Currently, there are no indications when surgery should be considered for asymptomatic, non-traumatic mesenteric hematoma. Herein, we report that collapse of a large mesenteric hematoma, which developed after rupture of a right colic artery aneurysm, resulted in small bowel obstruction and rapid absorption of the hematoma contributed to cholestasis. To our knowledge, complications due to the collapse of a mesenteric hematoma without re-rupture of the arterial aneurysm have not been previously described in the literature.

## Case presentation

**Fig. 1:**
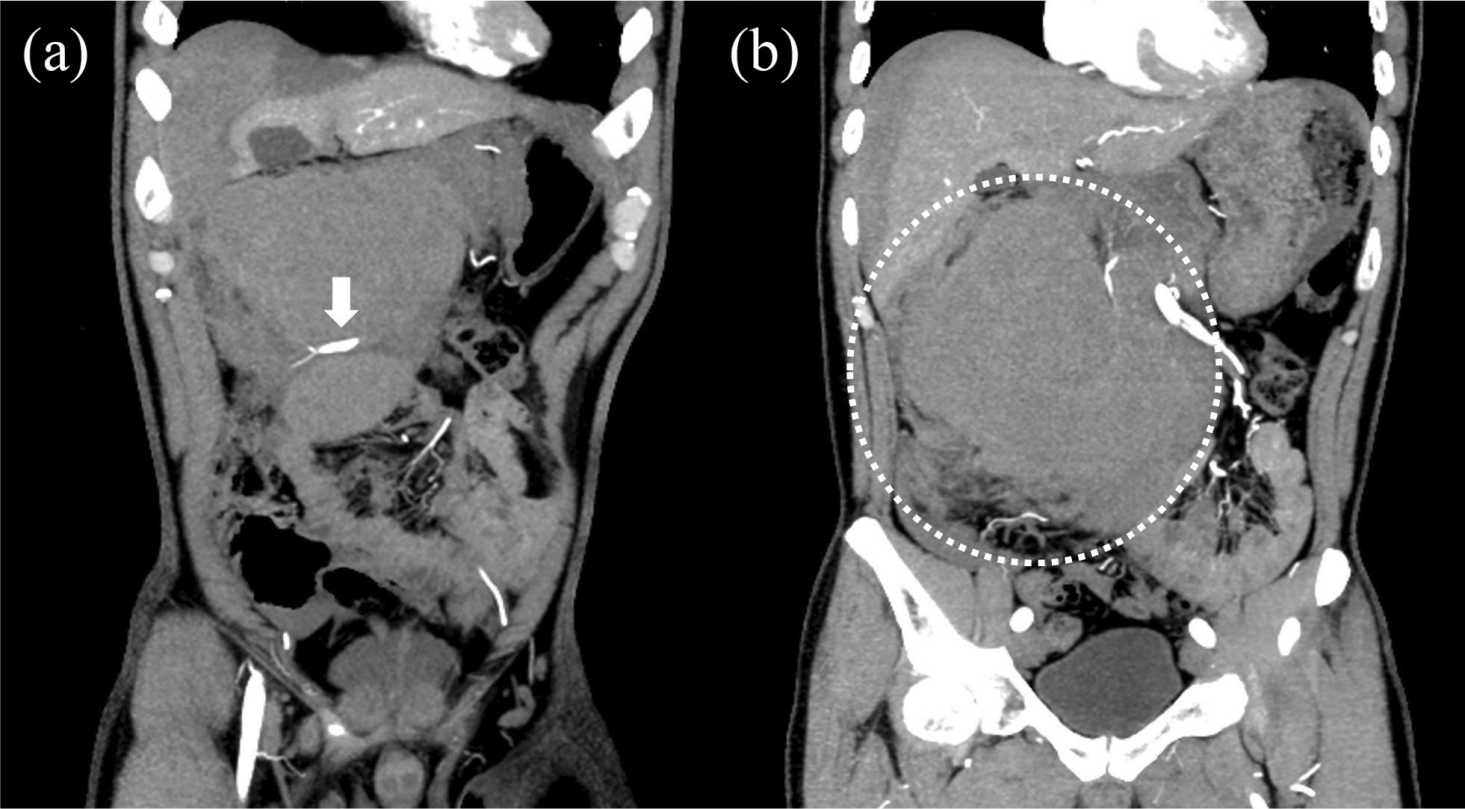
The computed tomography image shows the spindle-shaped aneurysm of the right colic artery (arrow) (**a**) and a large mesenteric hematoma (**b**)

**Fig. 2:**
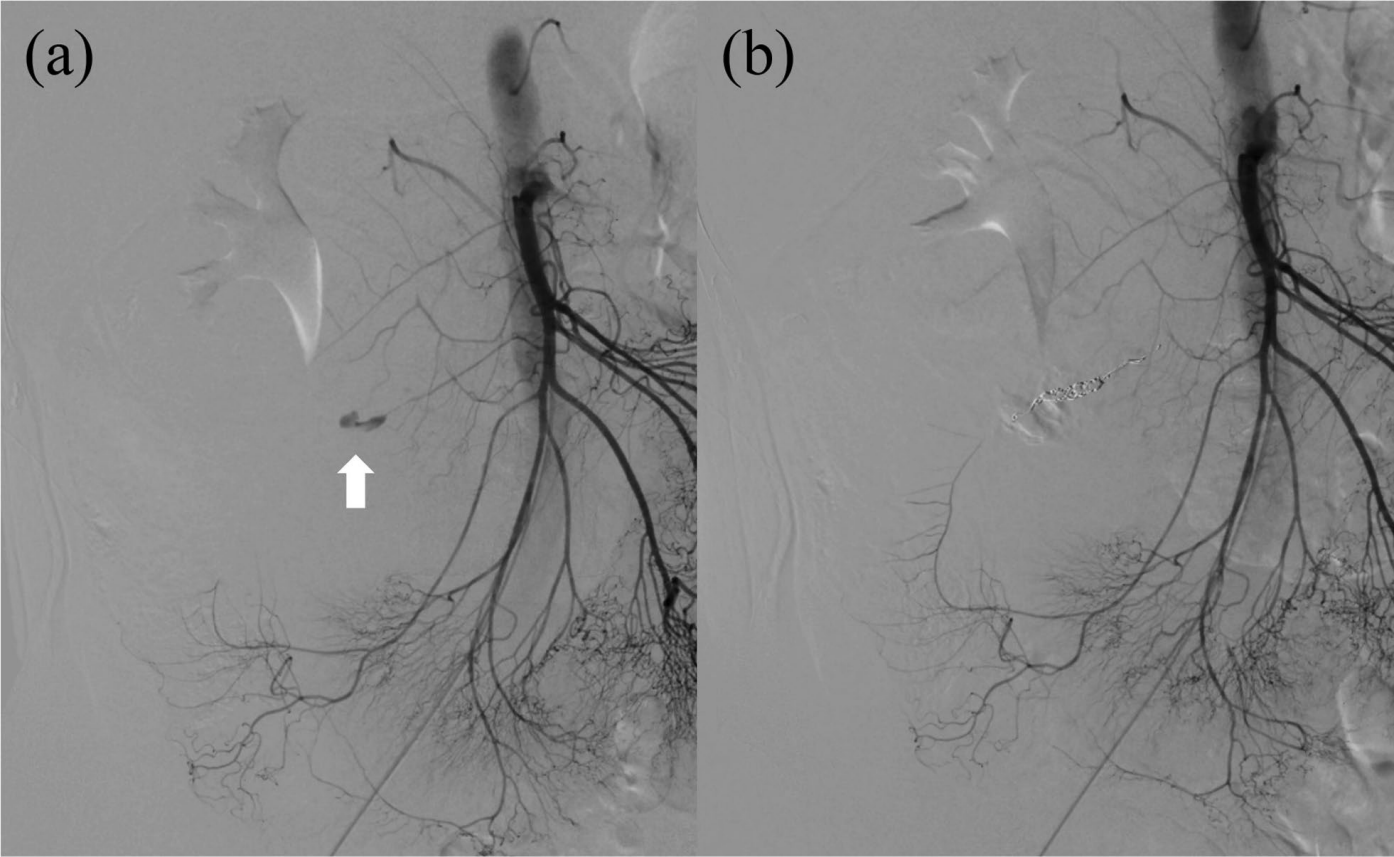
The angiography image shows the aneurysm of the right colic artery (arrow) (**a**) and coil embolization to the aneurysm (**b**)

**Fig. 3:**
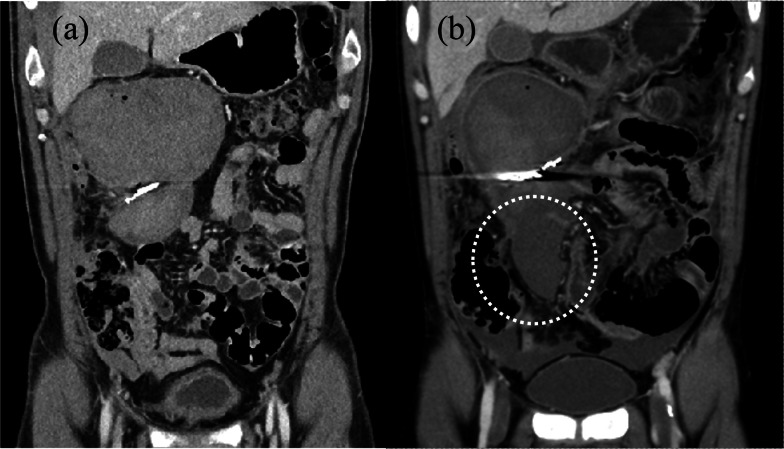
**A** Shows the hematoma after catheter intervention and **B** shows the collapse of the wall of the mesenteric hematoma, with leaking into the intra-abdominal cavity (round). A little air mixed in the hematoma during catheter intervention. The position and amount of air have not changed between both figures

**Fig. 4:**
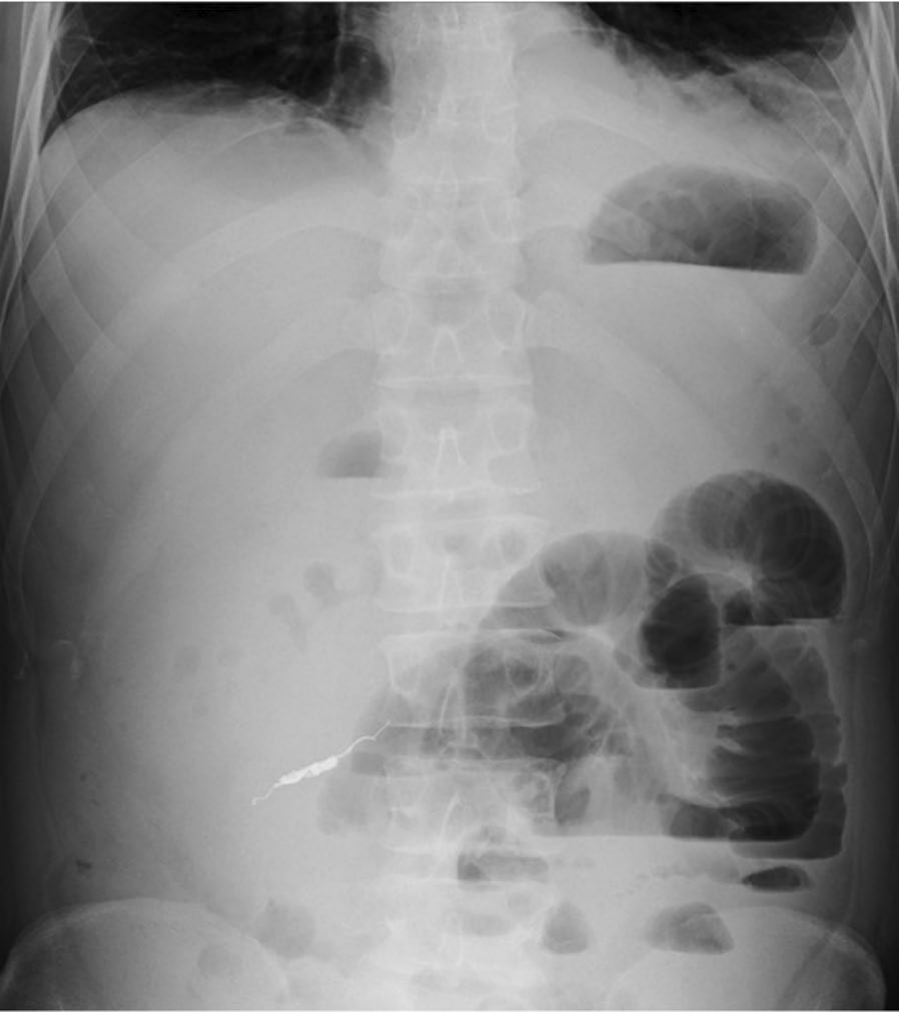
The X-ray shows the dilation and the niveau formation of the small bowel

**Fig. 5:**
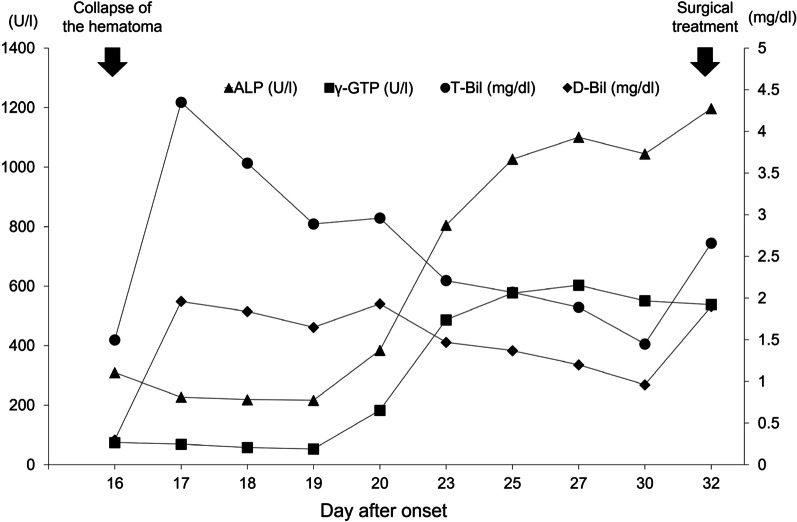
Clinical course of the levels of serum bilirubin and biliary enzymes prior to the surgical treatment. *ALP* alkaline phosphatase, *ɣ-GTP* gamma-glutaryl phosphatase, *T-Bil* total bilirubin, *D-Bil* direct bilirubin

A 44-year-old man was admitted to our hospital due to a sudden onset of lower right abdominal pain. The patient had a history of hypertension and hyperlipidemia. Computed tomography (CT) revealed a spindle-shaped and dilated right colic artery, intra-abdominal bleeding, and a large mesenteric hematoma (Fig. 1). A diagnosis was made of rupture of the aneurysm of the right colic artery, and catheter embolization of the ruptured right colic artery was performed (Fig. 2). Although cessation of bleeding was achieved, a large hematoma, with a major axis of 150 mm, remained in the ascending colic mesentery after catheter embolization (Fig. 3A). As the patient had no associated symptoms, he was discharged from the hospital on day 6 after onset. On day 16, however, the patient developed sudden onset of severe right lower abdominal pain. Although there was no evidence of re-bleeding on enhanced computed (CT) imaging, a collapse of the residual hematoma wall was observed, with visible leakage into the intra-abdominal cavity (Fig. 3B). Figure 3 shows a little air existed in the hematoma, although the position and amount of air have not changed between both Fig. 3A, B. Therefore, we considered that the air mixed during catheter and the hematoma did not have infection. After readmission, the patient developed a small bowel obstruction (Fig. 4), with an elevated level of biliary enzymes from day 20 (Fig. 5). CT and ultrasound images revealed dilation of the gallbladder and biliary sludge, but with no sign of inflammation or biliary obstruction. These findings were suggestive of cholestasis. Figure 5 shows the levels of serum bilirubin and biliary enzymes over this period. As the small bowel obstruction and cholestasis did not improve with conservative treatment, we proceeded to surgical treatment on day 32 after the initial onset.

### Operative findings

**Fig. 6:**
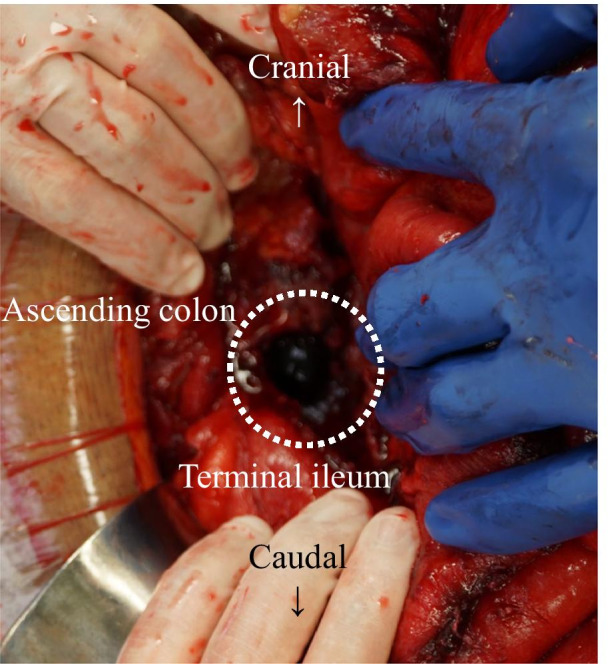
Intra-operative findings, showing the collapsed mesenteric hematoma (round), with adherence to the small intestine

We performed a laparotomy using a midline incision. Adhesions between the small bowel and the abdominal wall were observed, which were likely the cause of the small bowel obstruction. The release of these adhesions removed the cause of the small bowel obstruction. We also observed a collapse of the caudal wall of the hematoma in the ascending colic mesentery, with a pooling of the leaked blood in the pelvis (Fig. 6). We removed as much of the hematoma as possible and placed a drain in the pelvis and intra-mesenteric cavity. The culture taken from the hematoma during operation showed no growth of bacteria.

### Postoperative course

**Fig. 7:**
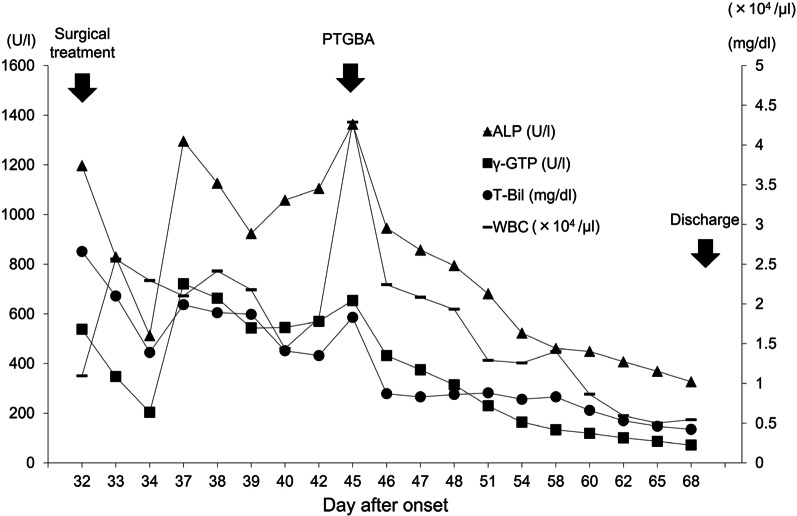
Clinical course of the levels of white blood cell count, serum bilirubin, and biliary enzymes after surgical treatment. *ALP* alkaline phosphatase, *ɣ-GTP* gamma-glutaryl phosphatase, *T-Bil* total bilirubin, *WBC* white blood cell count, *PTGBA* percutaneous transhepatic gallbladder aspiration

The small bowel obstruction improved after surgery and the patient was started on a diet. However, on day 45, the patient developed epigastric pain and fever. CT and ultrasound imaging revealed dilation of the gallbladder, with surrounding inflammation. From blood tests, the white blood cell count and levels of biliary enzymes and total bilirubin were elevated. The diagnosis of cholecystitis was made, and we proceeded with percutaneous transhepatic gallbladder aspiration (PTGBA). After PTGBA, the patient’s symptoms improved, with a decrease in white blood cell count and biliary enzymes and total bilirubin; the postoperative course of these levels is shown in Fig. 7. The patient was discharged on day 70 from the initial onset of symptoms, 38 days after surgical treatment.

## Discussion

Our case demonstrates the possibility of small bowel obstruction caused by the collapse of a mesenteric hematoma. This collapse caused leakage from the hematoma into the intra-abdominal cavity that allowed adhesions to form between the small intestine and the abdominal wall. The full diagnosis required diagnostic imaging, the patient’s clinical course, and intra-operative findings.

We identified five previous cases in the literature that reported complications due to a non-traumatic mesenteric hematoma without re-hemorrhage. The characteristics of these cases are summarized in Table 1 [3–7]. None of these cases reported complications due to the collapse of a large mesenteric hematoma, as occurred in our case after rupture of a right colic artery aneurysm. We note that our patient was asymptomatic until the partial collapse of the residual mesenteric hematoma wall on day 16 after the initial symptoms. Therefore, we consider that this collapse triggered the small bowel obstruction.

**Table 1:** Reported cases of complications of a non-traumatic mesenteric hematoma

Author	Etiology	Complication	Size (mm)	Treatment
Bekki et al. [3]	Unknown	Fistula between the transverse colon and the hematoma	120	Resection of the transverse colon
Shikata et al. [4]	Unknown	Fistula between the jejunum and the hematoma	30	Resection of the jejunum
Ashrafian et al. [5]	Crohn’s disease	Small bowel obstruction	200	Right hemicolectomy
Ono et al. [6]	Unknown	Duodenal stenosis	Not reported	Conservative treatment
Weinstock et al. [7]	Jejunal branch artery aneurysm	Small bowel obstruction	60	Gastrojejunostomy
Our case	Right colic artery aneurysm	Small bowel obstruction, cholestasis	150	Release of obstruction, reduction of hematoma

The intra-abdominal adhesions observed intra-operatively caused the small bowel obstruction after leakage of the hematoma into the intra-abdominal cavity. Previous studies have reported on small bowel obstruction [5, 7] or duodenal stenosis [6] caused by a non-traumatic mesenteric hematoma. In these cases, however, the obstruction was caused by direct compression of the gastrointestinal tract by the hematoma. In this sense, this would be similar to small bowel obstruction caused by blunt abdominal trauma causing mesentery injury and hematoma [8]. In contrast, our case reveals the possibility that collapse and leakage of the non-traumatic mesenteric hematoma might cause adhesions resulting in small bowel obstruction.

After the collapse of the mesenteric hematoma, our patient developed pooling of bile sludge in the gall bladder, and elevation of serum bilirubin and biliary enzymes before surgical treatment, followed by cholecystitis after surgery. We considered that bile sludge and the elevation of serum bilirubin and biliary enzymes indicated cholestasis. Figure 5 shows that the level of indirect bilirubin was significantly elevated in the early days after the collapse of the hematoma, and is directly associated with hematoma [9]. After the collapse, rapid absorption of the hematoma was reflected in the rapid elevation of biliary enzymes and cholestasis (Fig. 5). This finding is consistent with the report by Ichinomiya et al. [10] of cholestasis after abdominal trauma related to hematoma absorption. In our case, the presence of bile sludge, the elevation of biliary enzymes, and cholecystitis after surgery cannot deny the influence of small bowel obstruction and fasting, in addition to the collapse of the hematoma. However, it was previously reported that hemolytic disease promotes the formation of bile acid and stone [11, 12]. At least, we considered that the collapse of the hematoma might contribute to these conditions, including cholecystitis, through the mechanism of absorption of old hemolytic blood.

When the patient was discharged for the first time, we opted for conservative management as he was asymptomatic. This is consistent with the recommendations by Corzo et al. [13] for non-operative management of traumatic mesenteric hematoma when patients have no symptoms. They reported that the success rate of non-operative management was 100%. Therefore, conservative therapy is usually adequate for patients with no symptoms or physiological shock. However, their report did not clarify the relation between the size of hematoma and operative management. Moreover, Bekki et al. [3] reported a spontaneous mesenteric hematoma, with a major axis of 120 mm, caused by a fistula in the transverse colon, which developed during the period of observation and required resection. Ashrafian et al. [5] also reported a 200-mm hematoma that caused small bowel obstruction and required surgery. Based on these previous cases and our own, we propose that a large hematoma might be a risk factor for failure of conservative treatment. According to Table 1, including our case, any hematoma whose major axis was over 100 mm caused a fistula between the intestine and the hematoma or small bowel obstruction. Similar to our case, a large hematoma might be difficult to absorb, and that caused some complications. Therefore, we consider that surgery might be required when the major axis is longer than 100 mm. Thus, in the case of a large hematoma, we need to monitor the patients carefully despite the absence of symptoms. We should consider removing the mesenteric hematoma to avoid complications when the hematoma is not absorbed, or some symptoms appear, such as pain or tenderness.

## Conclusions

Collapse of a large mesenteric hematoma after rupture of a right colic artery aneurysm resulted in small bowel obstruction. Furthermore, the hematoma might contribute to cholestasis through the rapid absorption of the hematoma. Therefore, we considered that a large abdominal hematoma might be a risk factor for failure of conservative treatment, and surgical treatment might be required due to complications.

## Data Availability

Not applicable.

## Abbreviations

CT: Computed tomography
PTGBA: Percutaneous transhepatic gallbladder aspiration
